# Catalytic Effect of Hydrogen Bond on Oxhydryl Dehydrogenation in Methanol Steam Reforming on Ni(111)

**DOI:** 10.3390/molecules25071531

**Published:** 2020-03-27

**Authors:** Changming Ke, Zijing Lin

**Affiliations:** 1 Hefei National Laboratory for Physical Sciences at Microscales, Department of Physics, School of Physical Sciences, University of Science and Technology of China, Hefei 230052, Anhui, China; cmk@mail.ustc.edu.cn; 2 CAS Key Laboratory of Strongly-Coupled Quantum Matter Physics, Department of Physics, School of Physical Sciences, University of Science and Technology of China, Hefei 230052, Anhui, China

**Keywords:** Reaction mechanism, first-principle calculation, Bader charge analysis, activation energy, transition state structure

## Abstract

Dehydrogenation of H_3_COH and H_2_O are key steps of methanol steam reforming on transition metal surfaces. Oxhydryl dehydrogenation reactions of H*_x_*COH (*x* = 0–3) and OH on Ni (111) were investigated by DFT calculations with the OptB88-vdW functional. The transition states were searched by the climbing image nudged elastic band method and the dimer method. The activation energies for the dehydrogenation of individual H*_x_*COH* are 68 to 91 kJ/mol, and reduced to 12–17 kJ/mol by neighboring OH*. Bader charge analysis showed the catalysis role of OH* can be attributed to the effect of hydrogen bond (H-bond) in maintaining the charge of oxhydryl H in the reaction path. The mechanism of H-bond catalysis was further demonstrated by the study of OH* and N* assisted dehydrogenation of OH*. Due to the universality of H-bond, the H-bond catalysis shown here, is of broad implication for studies of reaction kinetics.

## 1. Introduction

Methanol steam reforming (MSR), H_3_COH + H_2_O → CO_2_ + 3H_2_, is frequently used for the generation of H_2_ at temperatures of 200–300 °C and atmospheric pressure [1,2,3,4]. MSR can be catalysed by a number of metals and metal oxides. Cu is the most common commercial catalyst but suffers from pyrophoricity and catalyst sintering, limiting its long-term applications [5,6]. Noble metals, such as Pd and Pt, that possess long term stability and no pyrophoric behavior [3,7] suffer from high price, limiting their large-scale industrial applications. On balance, Ni is a low price and highly effective MSR catalyst. The high activity of Ni for catalyzing MSR at T ≥ 300°C has been demonstrated in recent experiments [5,6], but a convincing theoretical explanation of the reaction mechanism is lacking.

There have been numerous theoretical studies on the reaction mechanism of MSR over a number of catalysts. Lin et al. conducted density functional theory (DFT) calculations and built a kinetic model of MSR on Cu(111) [8]. They found kinetic relevant steps with high activation energies include H_3_COH* + * → H_3_CO* + H* and, where * denotes an active surface site and R* means a surface adsorbed species R. Zuo et al. discussed the mechanisms of methanol decomposition, methanol oxidation and steam reforming of methanol on Cu(111) [9]. Wang et al. explained the differentiation of intrinsic reactivity of MSR on Cu, CuZn and Cu/ZnO [10]. In addition, they proposed a microkinetic model for more in-depth mechanics research of MSR on Cu [11]. Smith et al. conducted DFT studies on the initial steps of MSR on PdZn and ZnO surfaces, and found defect sites lower the barrier significantly [12]. Also based on DFT calculations, Krajčí et al. demonstrated the CO/CO_2_ selectivity of MSR on many alloys, e.g., PdZn, PtZn and NiZn [13]. Chen et al. [14], Lausche et al. [15], and Kramer et al. [16] studied the selectivity of the dehydrogenation of methanol on Cu(110), Ni(100) and Ni(111), respectively. MSRs on Pt [16], Pd [16], Pt_3_Ni alloy [17], Pt-Skinned PtNi Bimetallic Clusters [18] and Co [19] have also been investigated.

Summarizing the existing results of MSR studies, there are two kinds of MSR kinetics. The first was deduced by considering the dehydrogenation of isolate adsorbed H*_x_*COH (*x* = 3, 2, 1, 0). As the activation energy for the bond breaking of H_3_CO-H* is high, H_3_COH* + * → H_3_CO* + H* was found to be a likely rate determining step (RDS) [8,10,11,17]. For example, Wang et al. [11] showed in their DFT study that the activation energy for CH_3_OH* + * → CH_3_O* + H* was 103 kJ/mol and MSR on Cu(111) was mostly limited by the dehydrogenation of CH_3_OH*. The second considered the interaction of H_3_COH* and OH* where the co-adsorbed OH* significantly reduces the activation energy of H_3_COH* + OH*→ H_3_CO* + H_2_O* and the C–H scission steps were found to be rate limiting [9,13,19]. The resulting kinetics with a lower activation energy agrees better with the experiments [20,21], and is a clear improvement of the first one. Unfortunately, the improved understanding has so far been mainly limited to a computational detail based deduction. In-depth understanding based on general physical concept and/or generable mechanism is highly desirable.

This work focuses on the role of hydrogen bond (H-bond) on reducing the activation energies of oxhydryl dehydrogenation that are important for determining the kinetics of MSR on Ni(111), the dominant catalyzing surface of micron-sized commercial Ni catalysts [22]. DFT calculations were performed to examine the oxhydryl dehydrogenation of H*_x_*CO–H* (*x* = 3, 2, 1, 0), with and without the assistance of the co-adsorbed OH*. Combined analysis of Bader charges and transition state structures showed that H-bond is the root cause for the observed high MSR activity. The catalytic role of H-bond was further supported by investigating the dehydrogenation of O–H* assisted by co-adsorbed N*.

## 2. Computational Methods

DFT calculations were performed using Vienna Ab-initio Simulation Package (VASP) [23,24,25,26], a plane wave computational software. The projector augmented wave (PAW) method [27,28] was used to describe the electron-ion interaction between core ion and valence electrons. The Kohn-Sham equations were solved with a 380eV cutoff energy for the wavefunctions of valence electrons. The exchange correlation interaction was described by the functional of OptB88-vdW [29,30]. OptB88-vdW was chosen as it best describes the van der Waals (vdW) interaction on metal surface [31]. The computations were performed on a three-layer slab of 3 × 3 unit cell surface model of Ni(111), with a vacuum region of 10 Å thickness. The surface layer of the slab was allowed to relax, while the bottom two layers were fixed. Spin polarization and dipole correction were considered by setting SPIN = 2 and LDIPOL = .True in all calculations. The Brillouin zone was sampled by a 5 × 5 × 1 k-point Monkhorst-Pack grid. All stable structures were optimized with an energy-based conjugate gradient algorithm [32]. The convergence criteria for electronic and ionic energies were 10^−6^ eV/atom and 10^−5^ eV/atom, respectively. The cutoff energy and the k-point grid were tested to be appropriate, e.g., the differences in the obtained adsorption energy of H_3_CO are less than 0.4 kJ/mol and 0.7 kJ/mol when compared to a cutoff energy of up to 460 eV and a k-point grid of up to 8 × 8 × 1, respectively.

Saddle points were determined by combining the climbing image nudged elastic band (CL-NEB) method [33] and the dimer method [34]. First, the less computing intensive CL-NEB was used to find the minimum energy path and the transition state. In the CL-NEB calculations, 7 images were inserted between reactants and products, and the electronic energies and the forces were converged to 10^−4^ eV/atom and 0.03 eV/Å, respectively. Second, the transition states obtained by the CL-NEB searches were used as the inputs for the high-precision dimer method [34] to find the accurate transition states efficiently. In the dimer method calculations, the electronic energy and force were converged respectively to 10^−7^ eV/atom and 0.01 eV/Å.

The Bader charge was calculated by the method of partitioning charge density grids into Bader volumes, as proposed by Henkelman’s group [35,36].

## 3. Results and Discussion

### 3.1. Oxhydryl Dehydrogenation of HxCOH*

**Figure 1:**
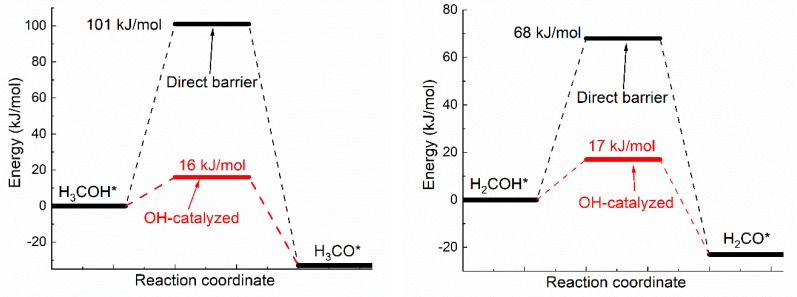

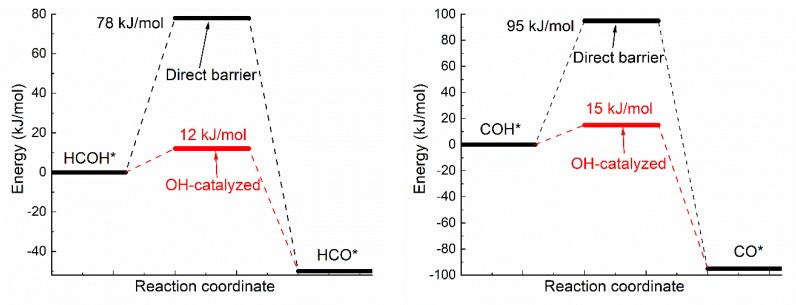
Activation energies of dual path dehydrogenation of oxhydryl in H*_x_*CO–H* (*x* = 3, 2, 1, 0).

Oxhydryl dehydrogenation of isolated H*_x_*COH* (*x* = 3, 2, 1, 0), H*_x_*COH* + * → H*_x_*CO* + H*, and that assisted by co-adsorbed OH, H*_x_*COH* + OH* → H*_x_*CO* + H_2_O*, were considered. The activation barriers of the two types of dehydrogenation reactions were compared in Figure 1.

Notice that the direct barrier of H_3_COH* dehydrogenation shown Figure 1 is about 91 kJ/mol. In comparison, the barrier was found to range from 39 to 75 kJ/mol in a few early studies [16,37,38]. The difference, due to various factors such as the use of different functionals, surface slab model and transition state search methods, is quite substantial, but is also seen in similar cases. For example, the direct barrier of H_3_COH* dehydrogenation on Cu(111) was found to vary from 62 to 138 kJ/mol by different DFT studies [8,9,10,11,39]. That is, the difference known for Cu(111) is comparable to that known for Ni(111). Although it is premature to draw any conclusion, the result here may be preferable due to the demonstrated quality of OptB88-vdW for similar systems [31] and the widely accepted slab model and transition state search method.

As shown in Figure 1, the activation energies (*E*_a_’s) of oxhydryl dehydrogenation of H*_x_*COH* are reduced from 68–91 kJ/mol for isolated H*_x_*COH* to 12–17 kJ/mol for H*_x_*COH* with co-adsorbed OH (*x* = 3, 2, 1, 0). The reduction of *E*_a_ for the oxhydryl dehydrogenation of H_3_COH* due to the presence of neighboring OH* is known in literatures. For example, *E*_a_ is reduced from 62 kJ/mol to 32 kJ/mol for Cu(111) [9] and from 80 kJ/mol to 22 kJ/mol for Co(111) [19]. The results here concerning the oxhydryl dehydrogenation of H*_x_*COH* for *x* = 2, 1, 0 indicate that the effect is quite general. The low activation energies mean that all oxhydryl dehydrogenation processes of H*_x_*COH* in MSR should be sufficiently fast. Besides, the energy cost for a close proximity of H*_x_*COH*and OH* as compared to isolated adsorbates is low, at 7.7, 14, 11, 5.1 kJ/mol for *x* = 3, 2, 1, 0, respectively. Therefore, there is no need to consider the oxhydryl dehydrogenation processes of H*_x_*COH* when examining the possible RDS in MSR. This result can be used to simplify the elementary reaction step study in many relevant problems. A low *E*_a_ for H_3_COH* dehydrogenation is also necessary for the understanding of the high MSR activities of Ni catalysts observed experimentally [5,6].

Notice that, while OH* facilitates the O–H scission process, OH* provides no help for C-H scission. The activation energies for CH_3_O* + OH* → CH_2_O* + H_2_O* and CH_3_OH* + OH* → CH_2_OH* + H_2_O* are 166 and 149 kJ/mol, respectively. Both the activation energies are higher than the corresponding activation energies of 86.8 and 91.5 kJ/mol for CH_3_O* → CH_2_O* + H* and CH_3_OH* → CH_2_OH* + H*, respectively. Similar result has also been observed for the C-H scission on PdZn(111) [40]. As reactions prefer the least resistant paths and the fractional coverage of OH* is in the order of 1% and very low coverages for CH_3_OH* and CH*_x_*O* on Ni(111) [41], the C–H scission is not expected to be adversely impacted by OH*. Due to the high *E*_a_ involved in CH_3_O* → CH_2_O* + H* or CH_3_OH* → CH_2_OH* + H*. However, the C–H scission step is expected to be rate limiting for MSR on Ni(111).

**Figure 2:**
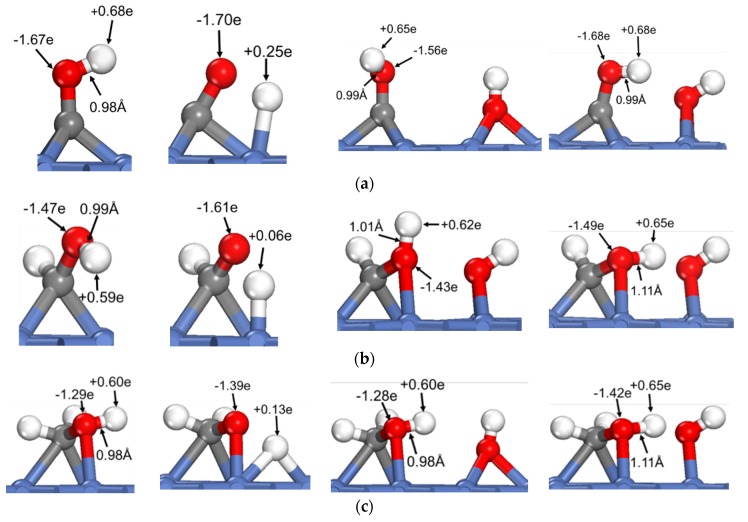

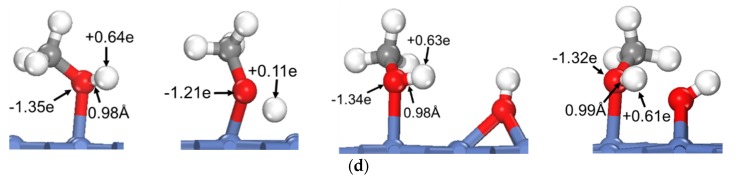
Structures and Bader charges in the oxhydryl dehydrogenation of H*_x_*COH with and without co-adsorbed OH: (**a**) *x* = 0, (**b**) *x* = 1, (**c**) *x* = 2, (**d**) *x* = 3. From left to right: local minimum and transition state structures of H*_x_*COH without and with co-adsorbed OH.

To reveal the common feature of oxhydryl dehydrogenation in different H*_x_*COH*, Figure 2 shows the structures and Bader charges of H*_x_*COH*, with and without OH* co-adsorption, at their initial local minimum geometries and reaction transition states. As seen in Figure 2, the Bader charges of oxhydryl H for isolated H*_x_*COH* at their local minimum and transition state structures are on average +0.63 e and +0.14 e, respectively. Clearly, the Bader charges of oxhydryl H of isolated H*_x_*COH* at their local minimum and transition state structures are quite different. That is, a significant charge density redistribution is required in the reaction path going from a local minimum energy structure to a transition state configuration. A large electronic energy changes due to the orbital reorganization, or correspondingly a high energy barrier, is expected in the O–H scission process. In comparison, the Bader charges for oxhydryl H of H*_x_*COH* with co-adsorbed OH* are on average +0.59 e and +0.65 e at the minimum energy and transition state structures, respectively. There are little charge redistributions required in the bond scission reaction paths. Combined with the fact that the O–H bond of H*_x_*COH* is floppy, a very low activation energy is encountered for each of the reactions.

Based on the above analysis, it is clear that OH* plays a catalyzing role in the oxhydryl dehydrogenation of H*_x_*COH* on Ni(111). The catalysis effect is realized by minimizing the charge redistribution requirement in the reaction path. The charge of oxhydryl H of H*_x_*COH* in the reaction process is maintained by interacting with OH*. The H⋯OH distance at the transition structure is 1.86, 1.61, 1.67 and 2.05 Å for *x* = 0, 1, 2, and 3, respectively. The distances are characteristics of H-bonds. Therefore, the reduced reaction barrier for the oxhydryl dehydrogenation of H*_x_*COH* can be attributed to the catalyzing effect of H-bond interaction.

### 3.2. Dehydrogenation of OH* Assisted by H-Bond of O–H⋯OH and O–H⋯N

**Figure 3:**
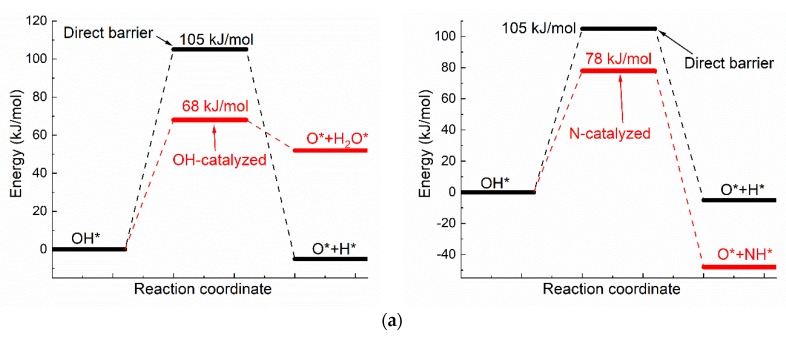

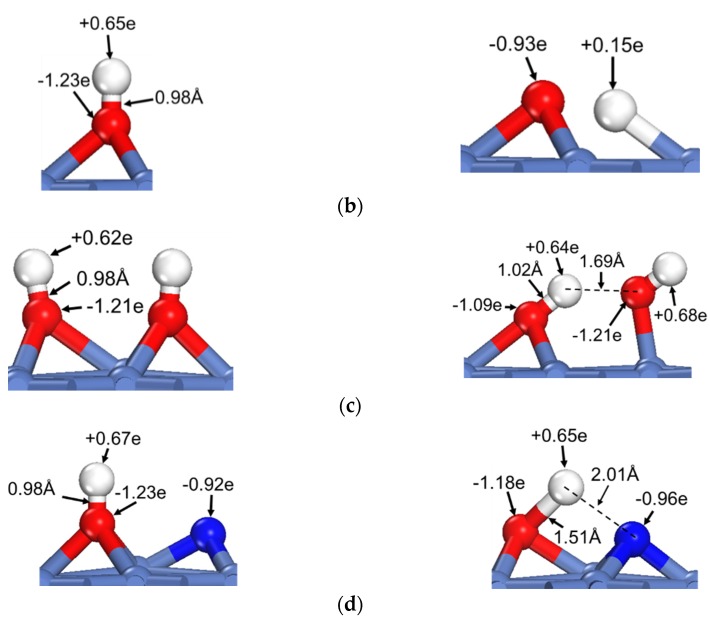
The dehydrogenation of O–H on Ni(111) surface: (**a**) The activation energies of dual path dehydrogenation of OH*, (**b**) The initial and transition state structures and charge distributions of individual OH* dehydrogenation, (**c**) The initial and transition state structures and charge distributions of OH-catalyzed OH* dehydrogenation, (**d**) The initial and transition state structures and charge distributions of N-catalyzed OH* dehydrogenation.

The catalyzing effect of H-bond on oxhydryl dehydrogenation can also be seen in the dehydrogenation of OH* adsorbed on Ni(111) [42]. Figure 3 compares the activation energies, initial and transition state structures and charge distributions of OH* dehydrogenations with and without co-adsorbed neighboring OH*. As seen in Figure 3, the activation energy of dehydrogenation is 105 kJ/mol for isolated OH*, but is reduced by 37 kJ/mol to 68 kJ/mol for OH* with co-adsorbed OH* due to the O–H⋯OH interaction. The reduction of activation energy is also observed for other transition metals. The activation energy for the corresponding reaction is reduced from 1.11 eV to 0.3 eV on Co(111) [19] and from 1.88 eV to 0.82 eV on Cu(111) [9]. Moreover, the higher energy of O* + H_2_O* in comparison with that of OH* + OH* is in qualitative agreement with both the theoretical and experimental results of Che et al [43].

Like the case shown in Figure 2, the Bader charges of H for the energy minimum and transition state structures in the dehydrogenation of individual OH* are quite different, at +0.65e and +0.15e, respectively. The corresponding Bader charges are respectively +0.62e and +0.64e in the dehydrogenation of OH* with the presence of the O–H⋯OH interaction. Once again, the charge of oxhydryl H remains almost constant in the reaction process due to the influence of the O–H⋯OH H-bond.

H-bonds are ubiquitous in nature and normally exist between electronegative atoms and H atoms covalently bound to similar electronegative atoms. In addition to the OH⋯O H-bond discussed above, OH⋯N is another type of commonly seen H-bond. Even though OH⋯N is not involved in MSR, it may exist in other reaction processes. To test the conceptual generality of H-bond catalysis, the effect of OH⋯N H-bond on OH* dehydrogenation is examined here.

The activation energy, initial and transition state structures and charge distributions of OH* dehydrogenations with neighboring N* are also shown in Figure 3. As shown in Figure 3, the activation energy of OH* dehydrogenation is reduced by 27 kJ/mol due to the presence of OH⋯N interaction. Like the cases shown for OH⋯O interactions, the charge of oxhydryl H changes little in going from the initial local minimum structure to the transition state, even though the direction and distance of the O–H bond are substantially changed in the process.

Combining Figure 2 and Figure 3, a general conclusion may be drawn: the charge of oxhydryl H is kept unchanged in the initial and transition states of dehydrogenation by the presence of H-bond. As a result, the activation energy for the dehydrogenation reaction is reduced in comparison to that in the absence of the H-bond.

It is worth noting that the activation energy is reduced to a very low value of around 15 kJ/mol, for the dehydrogenation of H*_x_*COH*, but to 68–78 kJ/mol for the OH* dehydrogenation. The notable difference is attributable to the rigidity of the O-H bond in the species: The O–H bond directionality is quite weak in H*_x_*COH*, while relatively strong in OH*. Another point to note is that the activation energy for the H⋯N assisted reaction is 10 kJ/mol higher than that of H⋯O assisted process, even though the H⋯N interaction is known to be stronger than the H⋯O interaction on average. There is no surprise, though, as a specific case does not correspond to the average. As shown in Figure 3, the charge of N* for the H-bond is only −0.92 to −0.96 e, while the charge of O for the H-bond is −1.21 e. Moreover, the H-bond distance of OH⋯N is larger than that of OH⋯O. Consequently, the OH⋯N H-bond here is weaker and less effective in reducing the activation energy than the OH⋯O H-bond. Nevertheless, it is interesting to see the relatively weak OH⋯N H-bond is very effective in maintaining the charge of H in the reaction path that the O-H bond is stretched from the initial 0.98 Å to 1.51 Å at the transition state. Overall, a near constant charge of H during the O–H scission process as maintained by the H-bond interaction is a common feature for the H-bond catalyzed reactions. The effectiveness of an H-bond on lowering the activation energy is, however, dependent on numerous factors, such as the H-bond strength, the O–H bond strength, and the surface material, and thus requires further studies.

## 4. Conclusions

Oxhydryl dehydrogenations of H*x*COH* (*x* = 3, 2, 1, 0) on Ni(111) were investigated by DFT calculations and Bader charge analysis. The activation energies are 68 to 91 kJ/mol for isolated H*x*COH* and much reduced to 12–17 kJ/mol if assisted by neighboring OH*. The catalyzing effect of OH* is attributed to the OH⋯O H-bond that maintains the charge of oxhydryl H in the O–H bond breaking process. The catalytic mechanism of H-bond is further supported by the results of OH* and N* assisted dehydrogenation of OH*. Due to the universality of H-bond, the catalytic mechanics revealed here are of broad implication to the study of reaction kinetics of many systems.
